# Ontogenetic shifts in the metabolomic profiles of *Battus polydamas* (Lepidoptera: Papilionidae: Troidini) Feeding on *Aristolochia gigantea*

**DOI:** 10.1007/s10886-026-01769-5

**Published:** 2026-10-19

**Authors:** Sofia Coradini Schirmer, Thomas Christopher Rhys Williams, Jefferson Brendon Almeida dos Reis

**Affiliations:** 1 Graduate Program in Ecology, Department of Zoology, Institute of Biological Sciences, University of Brasília (UnB), Brasília, DF Brazil; 2 Department of Botany, Institute of Biological Sciences, University of Brasília (UnB), Brasília, DF Brazil; 3 Graduate Program in Microbial Biology, Department of Phytopathology, Institute of Biological Sciences, University of Brasília (UnB), Brasília, DF Brazil

**Keywords:** Insect–plant interactions, Defensive metabolites, Metabolomics, Developmental stages

## Abstract

Insect development involves profound physiological reorganization that can alter the assimilation and allocation of metabolites across life stages. In herbivorous insects, these processes are tightly linked to diet-derived chemical exposure, yet how metabolic organization shifts throughout ontogeny remains poorly understood. Here, we investigated ontogenetic metabolomic variation in *Battus polydamas* feeding on *Aristolochia gigantea*, a system characterized by a specialized ecological interaction between insect and host plant. We examined how chemical profiles vary across larval, pupal, and adult stages, as well as in the host plant and larval frass, to determine whether metamorphosis is associated with stage-specific reorganization of the insect metabolome. Multivariate analyses revealed clear separation among sample types, and PERMANOVA confirmed significant differences among groups. Pupae and adult bodies showed the greatest similarity, whereas plant and frass formed distinct clusters. Wings exhibited a differentiated metabolomic profile. Molecular networking revealed a diverse set of primary and secondary metabolites, including carbohydrates, amino acids, phenolics, fatty acids, and sterol-related compounds. Nine metabolites differed significantly among plant, insect stages, and frass. Lipid- and sterol-related compounds showed stage-specific variation, particularly in pupae and wings, while phenolic derivatives and fatty acids distinguished insect tissues from plant and frass samples. Frass displayed marked chemical divergence, consistent with differential assimilation and metabolic processing. In summary, the metabolomic profile of *B. polydamas* is reorganized across ontogeny, reflecting stage-specific patterns of metabolite retention and transformation throughout development.

**Supplementary Information:**

The online version contains supplementary material available at https://doi.org/10.1007/s10886-026-01769-5.

## Introduction

Insects, especially herbivores, exhibit remarkable physiological and biochemical adaptations that enable them to tolerate, detoxify, and exploit the diverse array of primary and secondary metabolites present in their diets (Petschenka and Agrawal 2016; Beran and Petschenka 2022). Over lineages, some insects have developed mechanisms such as detoxification, metabolic transformation, selective sequestration, and targeted storage of plant compounds, which shape their survival and ecological success (Petschenka and Agrawal 2016; Dussourd 2017). These adaptations allow insects not only to tolerate chemically defended plants and use them as hosts for feeding, but also, in some cases, to sequester these plant metabolites for their own defense (see Heckel 2014 for a definition). Such chemical strategies, however, are not fixed; they shift across life stages as insects undergo major developmental transitions, altering digestive physiology, metabolic demands, and interactions with host-derived compounds (Grant 2007; Pentzold et al. 2014; Song et al. 2018).

Physiological adaptations in insects to plant metabolites can be arranged along a continuum of strategies that range from avoiding exposure to potentially toxic compounds to actively incorporating these metabolites into insect tissues for defensive or communicative functions (Trigo 2000; Petschenka and Agrawal 2016; Beran and Petschenka 2022). In this way, species differ not only in the degree to which they tolerate specific plant chemicals but also in the metabolic, behavioral, and morphological mechanisms they employ to cope with them (Dussourd 2017; Beran and Petschenka 2022). However, even within the same species, growing evidence suggests that the capacity to process, sequester, and deploy plant metabolites changes considerably throughout insect ontogeny (Jones et al. 2019; Zagrobelny et al. 2014; Agrawal et al. 2021). Developmental stage–specific changes in feeding behavior, metabolic activity, and tissue differentiation can modulate both exposure to and utilization of plant chemicals. Consequently, the interplay between plant-derived metabolites and insect physiology may vary across larval, pupal, and adult phases, influencing not only survival and defense but also ecological interactions and life-history trajectories (Agrawal et al. 2021; Heckel 2014).

Lepidoptera is an order of insects known for pronounced ontogenetic transformations (Meister et al. 2018; Gallon et al. 2024; Shao et al. 2024), for ecological interactions ranging from generalist to highly specific (Altermatt et al. 2011; Fontanilla et al. 2022), and for a chemical diversity that remains underexplored, especially in ecological contexts (Cao et al. 2020; Shao et al. 2024; Tian et al. 2025; Trigo 2000). Most species have a life cycle marked by physiological reconfigurations that alter morphology, behavior, and metabolic composition (Kirkbride-Smith et al. 2001; Jing et al. 2012, 2014). In herbivorous larvae, essential nutrients for development are obtained from host plants (Altermatt et al. 2011; Greeney et al. 2012; Fontanilla et al. 2022; Schirmer and dos Reis 2026). The metabolites ingested can modulate steroid hormone activities and, consequently, the developmental trajectory of these insects (Kirkbride-Smith et al. 2001; Jing et al. 2012, 2014; Silva-Brandão et al. 2025). Some species have also evolved the ability to sequester plant metabolites and use them in their own defense (Sime et al. 2000; Priestap et al. 2012; Fontanilla et al. 2022). Papilionids such as *Battus polydamas* (L.) (Lepidoptera: Papilionidae: Troidini), for example, accumulate toxic compounds produced by *Aristolochia* spp., including aristolochic acids (AA) and their derivatives (Sime et al. 2000; Pinto et al. 2009; Priestap et al. 2012; Morais et al. 2013). Thus, the chemical composition of plants directly influences the multitrophic interactions between these insects, their environment, and the associated species (de Morais et al. 1997; Nishida 2002; Zagrobelny et al. 2014; Erb and Robert 2016; Silva-Brandão et al. 2025).

In swallowtail butterflies associated with *Aristolochia*, these dynamics are particularly intricate due to the chemical diversity of aristolochiaceous compounds. Studies on *B. polydamas archidamas* show that its larvae selectively sequester aristolactams from *(A) chilensis*, retaining aristolactam-II while extensively metabolizing aristolactam-I (Uzúa et al. 2013). Aristolochic acids are also accumulated by certain Papilionidae, contributing to larval toxicity (Nishida 2002). However, despite the ability of *(B) polydamas* to absorb AAs from its host plants, experimental evidence reveals that AAs I and II alone are insufficient to deter key predators, suggesting that other plant-derived metabolites or insect-mediated transformations may play a central role in its chemical defense (Morais et al. 2013). This system thus exemplifies both the selectivity and the complexity underlying metabolite sequestration in herbivorous insects, while also highlighting a significant gap in understanding how these processes shift throughout development and how ontogenetic changes shape the emergence of defensive phenotypes.

Considering the profound physiological reorganization that characterizes holometabolous development and the ability of papilionids to process host-plant metabolites, we hypothesize that the metabolomic profile of *Battus polydamas* is dynamically reorganized across ontogeny in a stage-specific manner. Accordingly, (i) plant and frass samples should exhibit chemical signatures distinct from insect tissues, reflecting selective assimilation and metabolic processing; (ii) pupae and adults are expected to exhibit higher metabolic similarity, reflecting shared metabolic processes during metamorphosis; and (iii) adult wings should show a distinct metabolomic composition consistent with tissue specialization.

## Materials and Methods

### Study System

*Battus polydamas* is one of the 12 species within the genus *Battus* and is widely distributed throughout the Neotropics, ranging from the southern United States to Argentina (Tyler et al. 1994). Larvae feed exclusively on plants of the genus *Aristolochia* (Aristolochiaceae) (Tyler et al. 1994), which contain toxic compounds such as benzylisoquinoline alkaloids and aristolochic acids (Holzbach and Lopes 2010; Lopes and Humpfer 1997). These substances are sequestered by the larvae, rendering both caterpillars and adults unpalatable to potential predators (Pinto et al. 2011). Females lay eggs in clusters on several *Aristolochia* species, and larvae exhibit gregarious behavior during early developmental stages (Brown et al. 1981; Tyler et al. 1994).

**Fig. 1:**
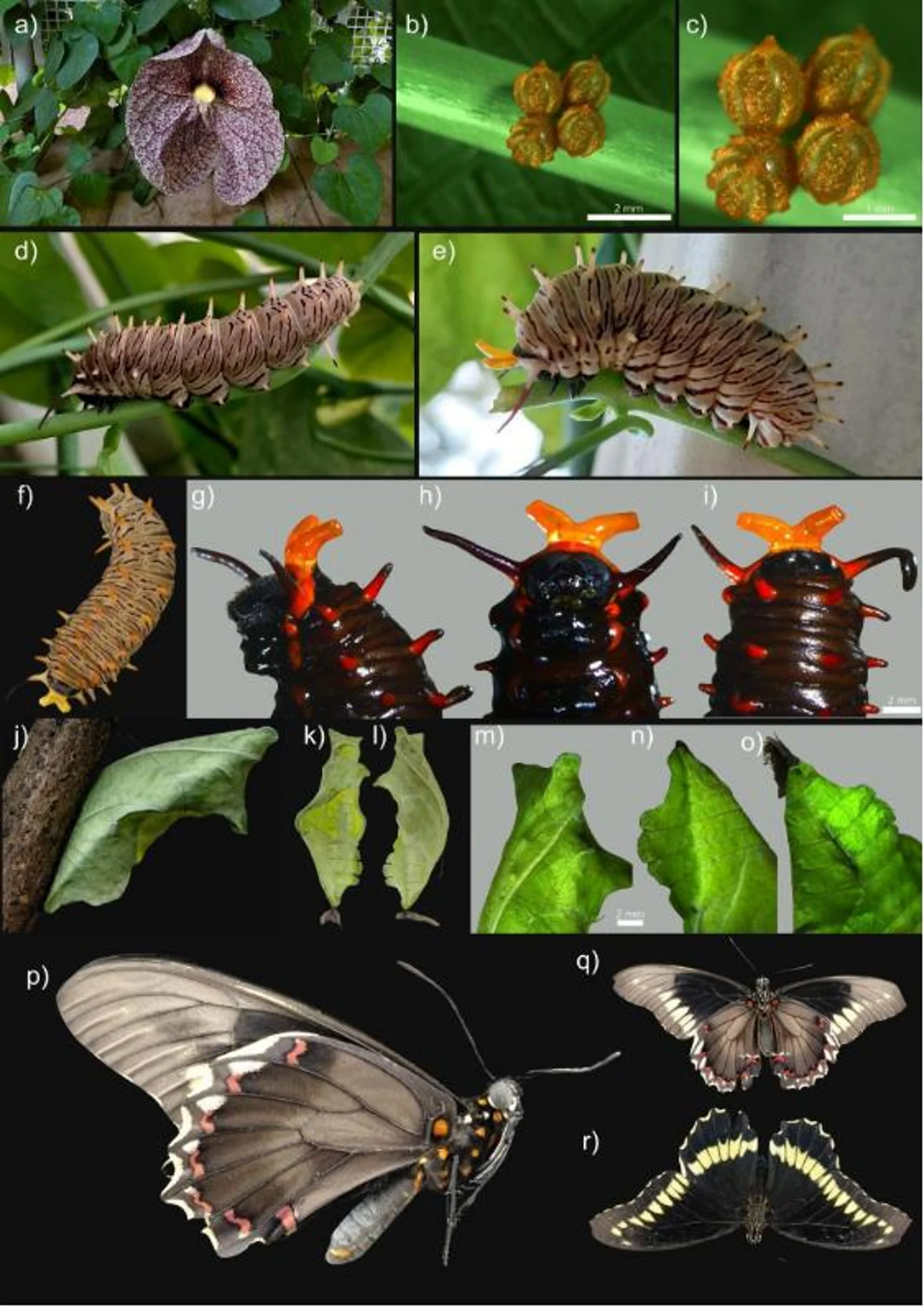
Morphological characteristics of *Aristolochia gigantea* Mart. & Zucc. (Aristolochiaceae) and the life cycle stages of *Battus polydamas* (Lepidoptera: Papilionidae: Troidini). (**a**) Fully opened flower of *(A) gigantea*, showing the reddish-purple limb with its characteristic reticulated pattern. (**b–c**) Newly deposited eggs of *(B) polydamas* attached to the host plant stem, arranged in compact clusters and exhibiting a textured, sculptured surface. (**d**) Mid-instar larva of *B. polydamas* on *(A) gigantea*, with a pale brownish body bearing dark longitudinal ridges and lateral tubercles. (**e–f**) Larvae displaying defensive behavior with evaginated, bright orange, bifid osmeterium. g–i) Lateral, frontal, and dorsal views of the everted osmeterium, showing its position and insertion on the prothorax. j–l) Pupae of *(B) polydamas* in different orientations, illustrating morphological variation in thoracic and abdominal contour and the cremaster used for attachment. (**m–o**) Dorsal and ventral details of the pupae, highlighting ridges, depressions, and the leaf-like venation pattern. (**p**) Lateral view of a freshly emerged adult, showing dark forewings and pink-orange abdominal markings. (**q**) Ventral view of an adult with contrasting cream-gray patterning on the hindwings. (**r**) Dorsal view of another adult, displaying the continuous yellow band on the hindwings characteristic of the species. The images were acquired using the protocol MSFP (dos Reis et al. 2025)

Small arthropods, including spiders and ants, are known predators of *B. polydamas* larvae (Brown et al. 1981; Morais et al. 2013). In addition to their aposematic coloration, larvae possess an osmeterium, a defensive eversible organ containing ellipsoid secretory glands (Fig. 1e–i) (Palma-Onetto et al. 2023). When disturbed, this organ can emit volatile compounds and may also function as a visual deimatic display to deter predators (Palma-Onetto et al. 2023).

### Plant Host Species

The family Aristolochiaceae is an early-diverging lineage of angiosperms, with species well-distributed throughout tropical and temperate regions (Kelly and González 2003). *Aristolochia* is the largest genus within this family, and contains more than 500 species (González 2012). Members of the genus *Aristolochia* are well known because of their peculiar flowers (Fig. 1a), which produce odours that primarily attract Diptera species (Sakai 2002; Aliscioni et al. 2017; Oelschlägel et al. 2015). *Aristolochia* contains aristolactams (ALs), a group of aporphinoids with a phenanthrene backbone in their structure (Wu et al. 2004), with some of these secondary metabolites being toxic to vertebrates (Wang et al. 2020). Despite these defenses, several specialist butterflies of the tribe Troidini use these plants as larval hosts (Tyler et al. 1994). *Aristolochia gigantea* was used as the host in this study. ALs and alkamides have been isolated from the stems of this species (Holzbach and Lopes 2010), whilst the leaves contain 8-benzylberbine-type alkaloids (Lopes and Humpfer 1997).

### Experimental Design

We collected 24 *B. polydamas* larvae at different larval stages from a single *(A) gigantea* plant located at the Institute of Biological Sciences of the University of Brasília, Darcy Ribeiro Campus (15°46′02.2"S; 47°51′50.8"W). A larger number of host plants could increase the sample size, but keeping all insects on the same plant allowed us to maintain the level of consistency required for meaningful comparisons. Similar approaches have been used in previous studies (Fontanilla et al. 2022). Since our goal was to investigate how the chemical profile changes throughout the insect’s ontogenetic stages, in its frass, and in its host plant, we used a single host plant species to minimize variation in the food source that could introduce confounding effects. This approach facilitated the identification of metabolic differences associated with the life cycle and trophic dynamics of *(B) polydamas.* This approach also ensures that all compounds present in the resource have a comparable probability of appearing at any trophic level, which is essential for interpreting these changes.

We analyzed six types of samples: larvae, pupae, adult bodies, adult wings, frass collected over seven days, and host plant leaves. We chose to separate adult specimens into wings and bodies to determine whether their chemical components are differentially distributed during the late developmental phase. We collected samples at different times to capture temporal variation in both the plant and the insects maintained on the same host individual. The larvae were transferred to acclimatized transparent containers and kept in a Biochemical Oxygen Demand (BOD) incubator (Percival Scientific Inc, I-37 LVL) incubator at 26 ± 2 °C, with controlled humidity between 70 and 80% and a photoperiod of 18 L:6D until they reached a size greater than 2.0 cm. After this point, they were maintained at room temperature (~ 22 °C) under a 12 L:12D photoperiod. Containers were cleaned and moistened daily, and the larvae were fed on newly expanded *A. gigantea* leaves, which we identified as the most commonly consumed leaf type of this species. Frass was collected individually from each larva in separate containers, ensuring that each individual represented an independent biological replicate and experimental unit. Samples were frozen immediately after collection and later weighed for metabolite extraction. Since larvae were collected directly in the field, precise determination of their instar was not possible; therefore, body size was used to standardize the start of frass collection and the time of euthanasia for individuals destined for extraction. After the seven-day collection period, larvae were fasted for 24 h and monitored for an additional six hours to confirm the complete cessation of frass production. This procedure ensured the clearance of plant-derived material from the digestive tract and prevented contamination of larval extracts with metabolites from the host plant (Fontanilla et al. 2022).

We maintained larvae that pupated at room temperature (approximately 22 °C) under a 12 h light/12 h dark photoperiod. We observed that the pupal period lasted between 18 and 21 days (*n* = 6). Based on this interval, we selected the tenth day after pupation for metabolite extraction, which corresponds to roughly half of the metamorphic process. Upon reaching adulthood, individual specimens were kept in the dark at 22 °C for 24 h after emergence to allow complete wing expansion and stabilization after eclosion, and then euthanized by freezing (*n* = 6). Before extraction, we separated the body from the wings using a sterile scalpel and processed each structure independently. For the host plant, approximately 15 fully expanded but still young leaves were collected at three separate time points spaced seven days apart, and each set of 15 leaves was treated as a sampling unit. Three sampling units per developmental stage (three individuals) were used for the insect samples due to space constraints and equipment availability. All samples were stored in a − 40 °C freezer until further processing and chemical analyses.

### Metabolite Extraction Protocol

We used three individuals from each developmental stage of *B. polydamas* for metabolite extraction. For the plant material, fifteen leaves were included per sampling unit. For the frass samples, all material produced over seven days was pooled and homogenized, and a 5 g subsample was used for metabolite extraction. We ground all samples in a pre-chilled mortar with liquid nitrogen until we obtained a fine and homogeneous powder. The material was transferred to chilled Erlenmeyer flasks, to which 15 mL of a mixture consisting of ethyl acetate and methanol (2:1, v: v) was added. This combination of solvents was used to enable extraction of metabolites with a wide range of different polarities (Ribeiro de Souza et al. 2009). The suspensions were then maintained for 24 h at room temperature. Next, we centrifuged the samples (9,000 rpm; 10 °C; 10 min), collected the supernatant, and evaporated the solvent under laminar flow until complete dryness. The dried extracts were weighed using an analytical balance and dissolved in ethyl acetate at 1 mg mL⁻¹. Samples were filtered through 0.22-µm hydrophobic PTFE membranes, transferred to 1.5 mL microtubes, and dried in a SpeedVac for 6 h prior to GC–MS derivatization and analysis.

### Gas Chromatography–mass Spectrometry (GC–MS) Metabolite Analysis

For gas chromatography–mass spectrometry (GC–MS) analyses, each dry extract was first derivatized with 40 µL of methoxamine hydrochloride (20 mg mL⁻¹ in pyridine) for 2 h at 37 °C and subsequently with 70 µL of MSTFA (N-trimethylsilyl-N-methyltrifluoroacetamide) for 30 min at 37 °C (Cury et al. 2020). Analyses were performed using an Agilent 7890 A gas chromatograph coupled to a 5975 C mass spectrometer (Agilent Technologies, Santa Clara, CA, USA), equipped with an HP-5MS capillary column (30 m × 0.25 mm × 0.25 μm; Agilent J&W Scientific, Folsom, CA, USA) (Cury et al. 2020). Samples were injected (1 µL) in split mode (30:1 split ratio), with the injector maintained at 230 °C. Chromatographic separation was performed under a constant helium flow of 0.6 mL min⁻¹. The oven temperature program began at 70 °C for 5 min, increased to 350 °C at 5 °C min⁻¹, and then decreased to 320 °C at 70 °C min⁻¹, where it was held for 5 min. Electron impact ionization was operated at 70 eV using a single quadrupole mass analyzer. Mass spectra were acquired in full-scan mode with a source temperature of 230 °C, quadrupole temperature of 150 °C, and transfer line temperature of 250 °C. The acquired spectra covered an ion range from *m/z* 60 to 549.9, with an average scan event time of approximately 0.337 s (≈ 2.97 scans s⁻¹), as retrieved from the raw NetCDF files. Two positive controls were included: chloramphenicol (1 mg mL⁻¹) and streptomycin (1 mg mL⁻¹). A solvent blank containing only ethyl acetate was also analyzed. Sample injection order was arranged in a random order to minimize run-order effects (dos Reis et al. 2024).

### Data Processing

Preliminary spectral inspection was performed using NIST (Gaithersburg, Maryland), whereas putative metabolite annotation used throughout downstream analyses was based on the GNPS GC workflow (https://ccms-ucsd.github.io/GNPSDocumentation/gcanalysis/). Chromatographic alignment and peak detection were performed using MetAlign (Lommen 2009). The software automatically corrected retention time shifts among chromatograms and generated an aligned feature matrix containing m/z values, aligned retention times, and peak intensities for all detected features. Raw data files (.CDS) were also inspected in MZmine 3 (Katajamaa et al. 2006; Heuckeroth et al. 2024) to visualize chromatograms and verify peak quality. The output file generated after alignment in MetAlign (Lommen 2009), containing peak intensities (m/z; rt) for all detected features, was used for downstream and multivariate analyses in MetaboAnalyst 6.0 (Pang et al. 2024). The data were uploaded and subjected to an initial consistency check. Duplicate features and those with more than 1% missing values were removed. We then applied a series of filtering steps to reduce instrumental noise and eliminate variables with limited informational value. A low-variance filter (Interquartile Range, IQR: 50%) was used to exclude features that were nearly constant across all conditions, and a low-abundance filter (mean intensity threshold: 30%) was applied to remove variables with intensities close to baseline. After filtering, the data were normalized using sample normalization by sum, log transformation (base 10), and auto-scaling. The processed dataset was then exported and used for multivariate analyses.

### Multivariate Analysis

The exploratory stage included unsupervised multivariate analyses (Worley and Powers 2013). Because one of our objectives was to assess similarities and differences among the chemical profiles of the distinct ontogenetic stages of *B. polydamas*, its frass, and its host plant (*A. gigantea*), we constructed a dendrogram using Euclidean distance and Ward’s clustering algorithm for hierarchical cluster analysis (HCA) (Răchieriu et al. 2021; Zelentsova et al. 2022). Principal component analysis (PCA) was used to investigate global variation patterns and identify natural groupings among the six sample types (Khadka et al. 2019). Additionally, heatmaps were generated to explore intensity differences and detect subsets of metabolites displaying distinct patterns across groups (Khadka et al. 2019). For this purpose, hierarchical clustering was performed at the feature level, grouping the GNPS-annotated metabolites according to similarities in their abundance patterns across samples, while sample-level clustering was assessed separately through HCA and PCA based on the overall chemical profiles.

### Library Search and Molecular Networking (GC Workflow)

Metabolite annotation was performed using the MSHub-GC workflow and Feature-Based Molecular Networking (FBMN) within GNPS (https://ccms-ucsd.github.io/GNPSDocumentation/gcanalysis/). Raw GC–MS data were subjected to automated spectral deconvolution, retention time alignment, and generation of a feature abundance matrix. Putative identifications were obtained by matching EI spectra against GNPS-integrated libraries, including MoNA, FiehnLib, the Borisov compilation, HMDB, and VOCBinBase. Molecular networking was conducted using the GNPS GC workflow. Spectra were filtered by removing fragment ions within ± 17 Da of the precursor m/z and retaining the six most intense ions within ± 50 Da windows. Mass tolerances were set to 20 Da (precursor) and 0.5 Da (fragment). Edges were established with cosine scores > 0.7 and at least six matched peaks, retaining only reciprocal connections among the top ten most similar nodes. Molecular families were limited to 100 nodes, and lower-scoring edges were removed iteratively. Library spectra were filtered under the same criteria, and matches were accepted with cosine > 0.5 and ≥ 6 matched peaks. Networks were visualized in Cytoscape (v3.7.2).

### Statistical Filtering and Downstream Analyses of GC–MS Molecular Networking Data

The node table generated by the Library Search/Molecular Networking (GC) workflow in GNPS was imported into R studio (v. 4.4.1) for processing and statistical analyses. Initially, only features that had been putatively identified based on an MQScore ≥ 0.75 and at least 10 shared fragment peaks were retained (Supplementary Material 2 - Raw Data; Filtered Data), ensuring greater reliability of the spectral annotations (Hahn et al. 2020; Liu et al. 2022). Unidentified entries were removed. To reduce the influence of analytical artifacts, compounds associated with recurrent contaminants and derivatization, such as siloxanes, trimethylsilyl derivatives, phthalates, and long-chain hydrocarbons, were excluded based on inspection of the assigned names (Hahn et al. 2020). The resulting dataset was used for subsequent analyses. Univariate comparisons were performed separately for each compound. Variance among groups was first assessed. If any group presented zero variance, normality could not be assessed, and the data were therefore considered not normally distributed. Otherwise, normality was evaluated using the Shapiro-Wilk test within each group, and homogeneity of variances was assessed using Levene’s test. Depending on which assumptions were met, one-way ANOVA was applied when normality and homoscedasticity were satisfied, Welch’s ANOVA was used when normality was present but variances were unequal, and the Kruskal-Wallis test was applied when normality was not met (Zhou et al. 2023). P-values were adjusted using the Benjamini-Hochberg method to control the false discovery rate. Compounds with an adjusted p-value < 0.05 were considered significantly different among groups. For significant compounds, multiple comparisons were performed according to the global test employed. Tukey’s HSD test was applied after ANOVA, while Dunn’s test with Benjamini-Hochberg correction was used after non-parametric analyses. Pairwise differences with an adjusted p-value < 0.05 were considered statistically significant (Zhou et al. 2023). Additionally, annotated metabolites that remained after filtering were subjected to metabolite set enrichment analysis in MetaboAnalyst 6.0 (Pang et al. 2024) using the enrichment analysis module with the “*pathway-based*” approach, applying the “*Gut Bacteria + Host reference library*,* which integrates 139 metabolic pathways annotated in KEGG*”.

## Results

### Data Inspection and Identification of Metabolite Profiles

**Fig. 2:**
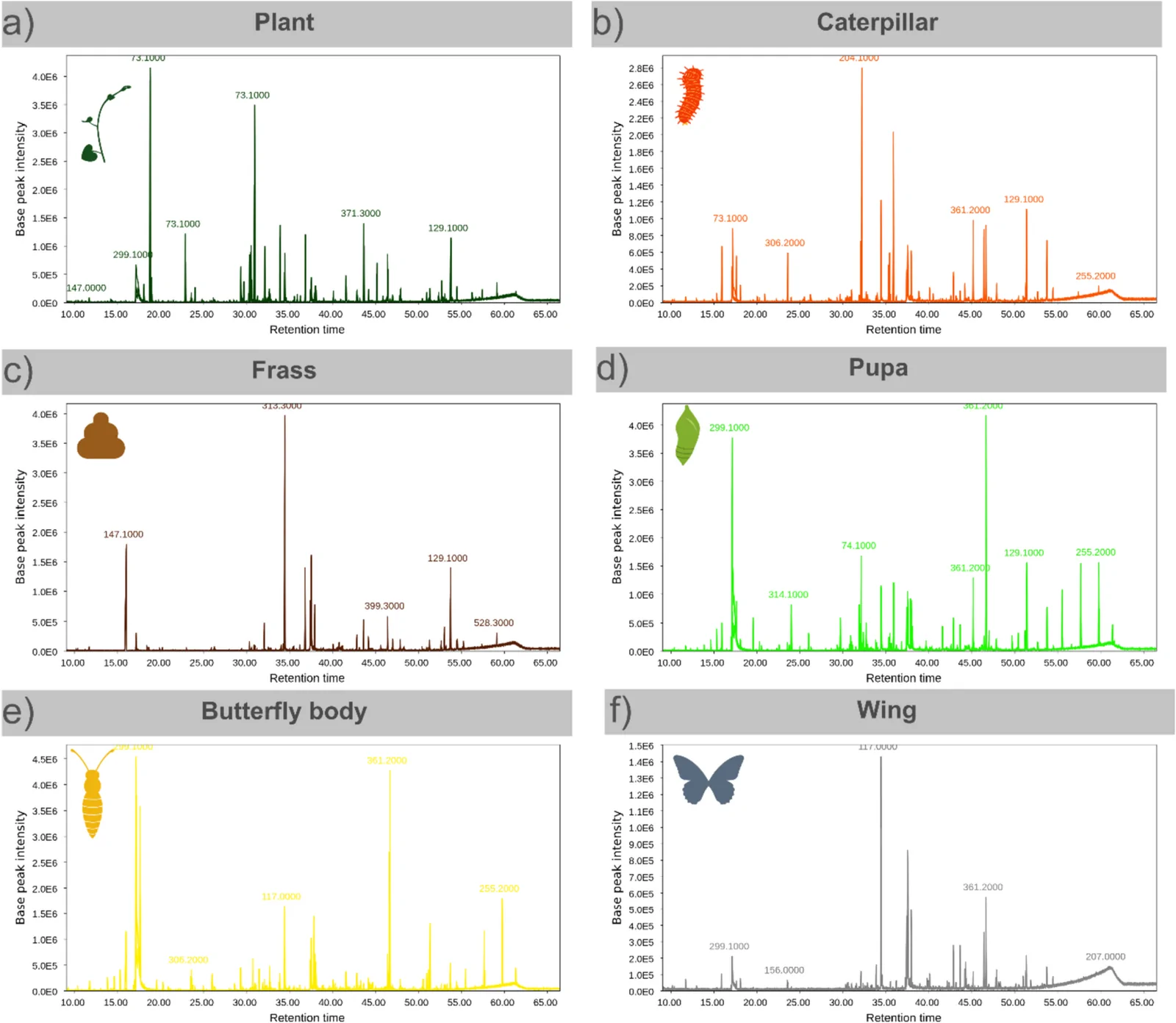
Total ion chromatograms (TICs) of the GC–MS extracts processed and visualized in MZmine 3.0. Chromatograms represent the chemical profiles of the six sample types analyzed: (**a**) *Aristolochia gigantea* leaves (Plant), (**b**) *Battus polydamas* larvae (Caterpillar), (**c**) larval frass (Frass), (**d**) pupae (Pupa), (**e**) adult body (Butterfly body), and (**f**) adult wings (Wing)

**Fig. 3:**
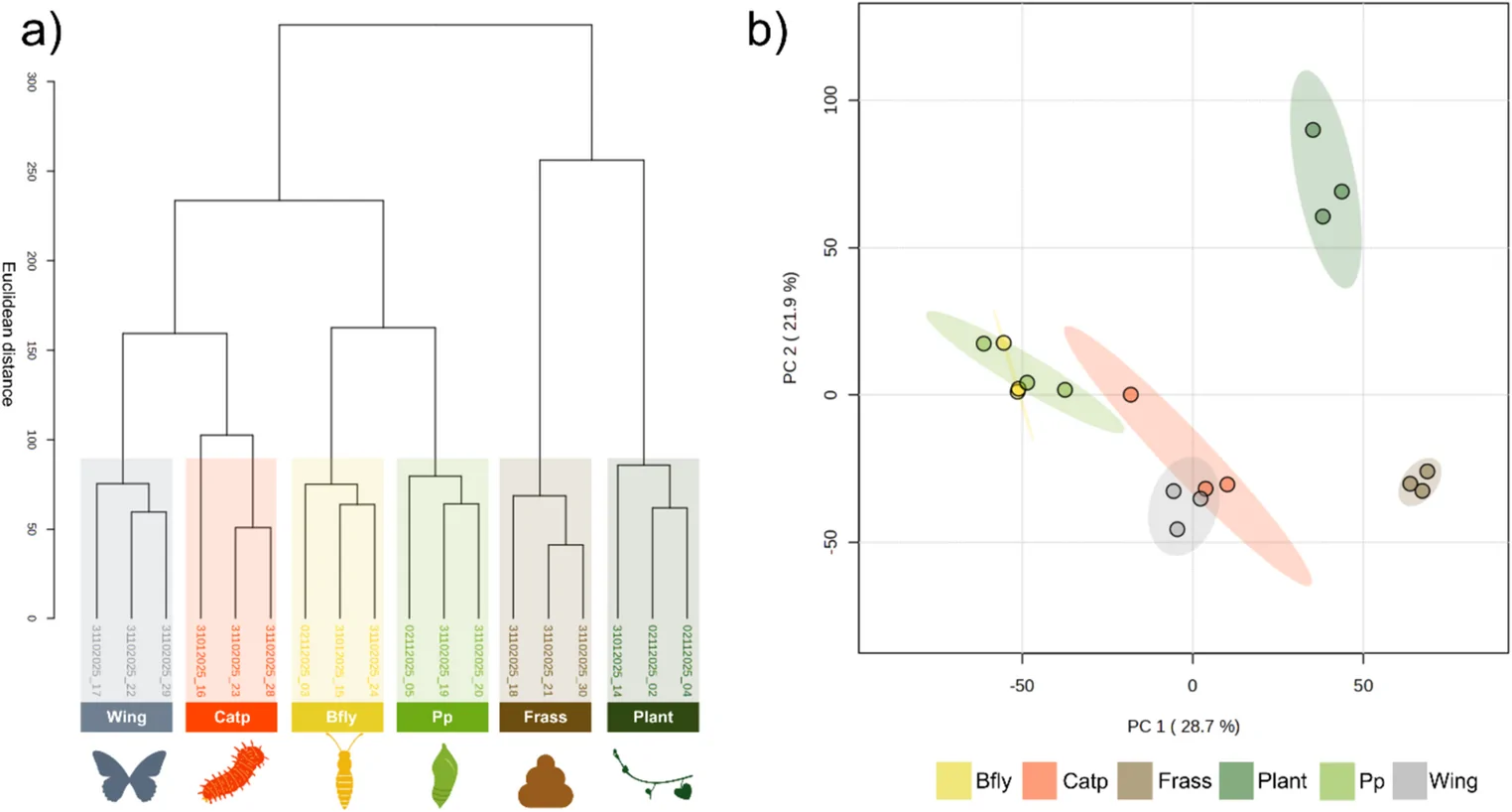
Exploratory analysis of the chemical profiles (GC–MS EI) of different developmental stages of *Battus polydamas* (caterpillar, pupa, and adult), along with frass and leaves of the host plant *Aristolochia gigantea*. (**a**) Hierarchical clustering dendrogram based on Euclidean distance. (**b**) Principal Component Analysis (PCA) score plot. Samples are projected onto the two-dimensional space defined by the first two principal components (PC1 and PC2), which together explain the majority of the observed variance. Bfly: Butterfly Body; Catp: Caterpillar; Frass: frass; Plant: Plant; Pp: Pupa; and Wing: Wing

GC–MS chromatograms revealed qualitative and quantitative differences among plant tissue, larvae, frass, pupae, adult bodies, and wings (Fig. 2). Hierarchical clustering based on Euclidean distances showed distinct relationships among the six sample types (Fig. 3a). At a lower clustering level, wing and caterpillar samples were grouped together, whereas butterfly body and pupa samples formed another cluster. Frass and plant samples remained separated from the insect-derived samples. At a higher clustering level, the insect-derived samples formed a single major cluster, clearly separated from plant and frass samples. PCA provided a complementary view of these relationships (Fig. 3b). PC1 and PC2 explained 28.7% and 21.9% of the total variance, respectively, accounting for 50.6% of the variation captured by the first two components. Plant and frass samples were strongly separated from the insect-derived samples, whereas butterfly body, pupa, and caterpillar samples occupied relatively close positions in the ordination. Wing samples were more clearly separated from the other insect tissues in the PCA, indicating differences in their overall metabolic profiles that were less evident in the hierarchical clustering. PERMANOVA confirmed significant differences in metabolic composition among sample types (F = 58.894; R² = 0.96084; *P* = 0.001).

### GC-MS and Feature Based Molecular Network Analysis

The molecular network constructed using MSHub-GC and FBMN comprised 519 nodes and 853 edges. The network presented an average of 4.857 neighbors per node and a density of 0.050 (Supplementary Material 2 – Supplementary Fig. 1). Topological parameters included a network diameter of 12, radius of 6, and a characteristic path length of 5.208. The clustering coefficient was 0.482, with network heterogeneity and centralization values of 0.679 and 0.065, respectively. A total of 99 connected components were identified.

**Fig. 4:**
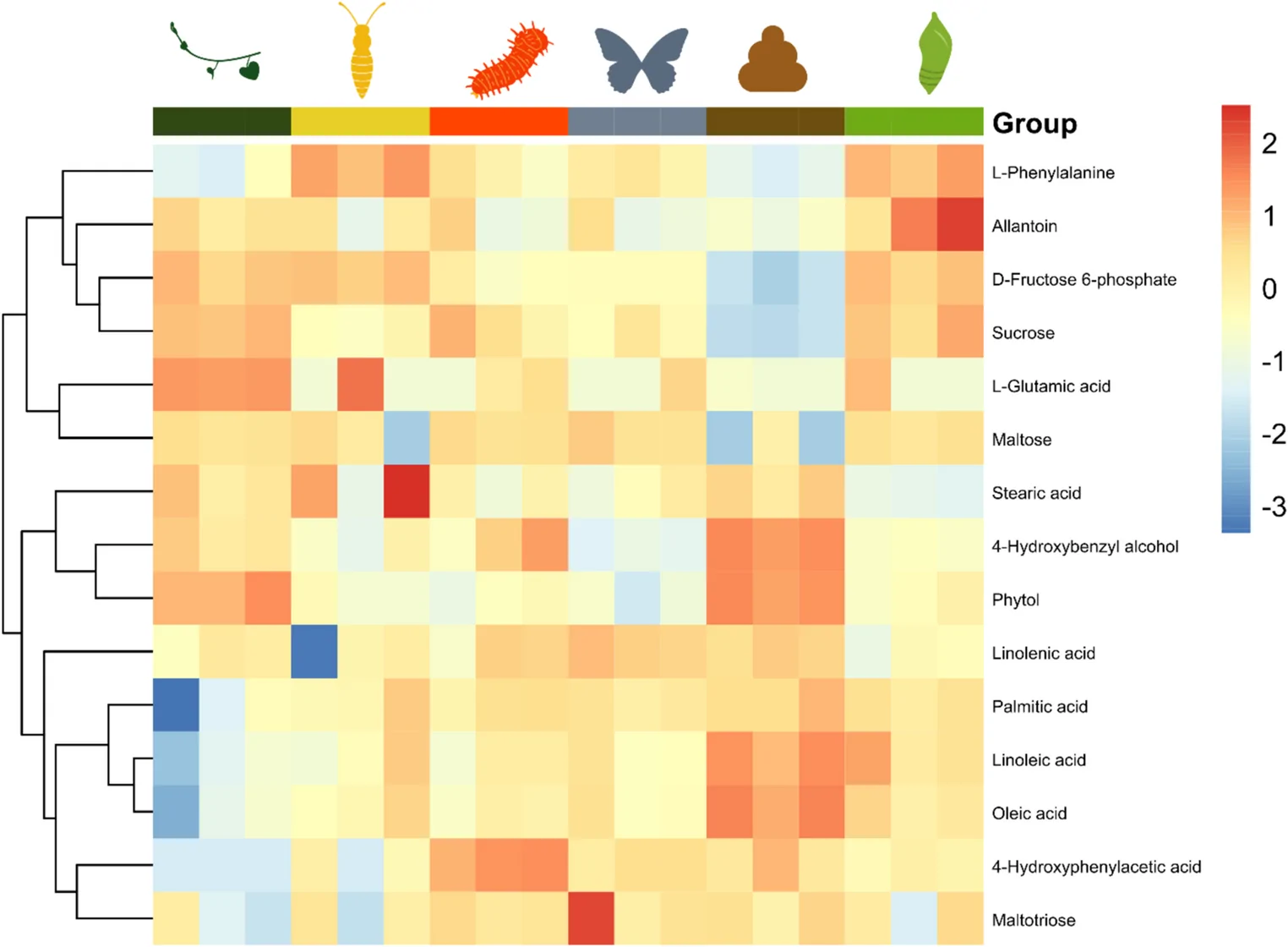
Heat map of annotated metabolites using molecular networks integrated into the GNPS workflow for GC MS EI data between developmental stages of *Battus polydamas* (caterpillar, pupa, butterfly body and wing), frass and leaves of the host plant *Aristolochia gigantea.* Data were autoscaled (z-score), and hierarchical clustering was performed at the feature level using Euclidean distance and Ward’s method to group metabolites according to similarities in their abundance patterns across samples. Rows represent metabolites and columns represent samples. Colors indicate relative abundance (blue = lower, red = higher intensity). Bfly: Butterfly body; Catp: Caterpillar; Frass: Frass; Plant: Plant; Pp: Pupa; Wing: Wing

The chemical characterization of the samples revealed a diverse metabolite profile (Fig. 4). Compound annotation reliability was supported by spectral similarity against GNPS libraries (average MQScore > 0.75) combined with detailed inspection of EI fragmentation patterns (Supplementary Material 1 – Filtered Data; Supplementary Material 2 – Supplementary Tables 1 and Supplementary Figs. 2–16). Detected compounds included carbohydrates such as maltose, sucrose, maltotriose, and D-fructose-6-phosphate; amino acid-related compounds including L-glutamic acid, L-phenylalanine, and allantoin; phenolic and aromatic compounds such as 4-hydroxyphenylacetic acid and 4-hydroxybenzyl alcohol; and fatty acids including palmitic acid, stearic acid, oleic acid, linoleic acid, and linolenic acid.

### Metabolites Significantly Differing Among Plant, Insect Stages, and Frass

**Fig. 5:**
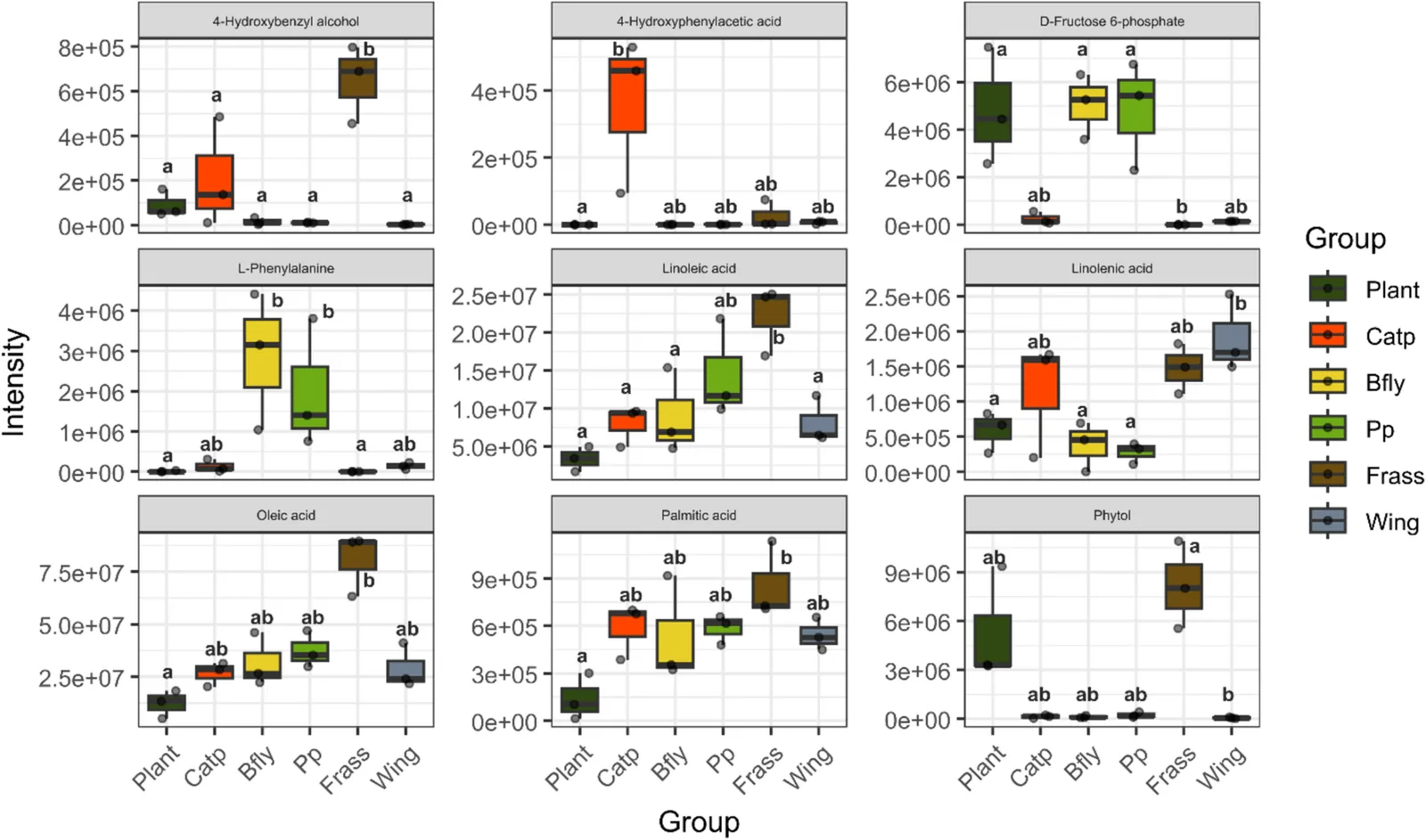
Boxplots of metabolites showing significant differences among developmental stages of *Battus polydamas*, frass, and *Aristolochia gigantea* leaves. The center line represents the median, the boxes indicate the interquartile range (IQR), the whiskers extend to 1.5 × IQR, and points beyond the whiskers represent outliers. The y-axis shows the relative abundance (peak intensity) of each metabolite. Different letters indicate statistically significant differences among groups (Tukey or Dunn post hoc tests, adjusted *p* < 0.05). Bfly: Butterfly body; Catp: Caterpillar; Frass: Frass; Plant: Plant; Pp: Pupa; Wing: Wing

Nine annotated metabolites showed significant differences among the plant, *Battus polydamas* developmental stages, and frass (Fig. 5). 4-Hydroxybenzyl alcohol differed between frass and all other groups (plant, caterpillar, butterfly, pupa, and wing; p.adj = 0.000456–0.0112). 4-Hydroxyphenylacetic acid differed between plant vs. caterpillar (p.adj = 0.0124). D-fructose 6-phosphate differed between frass vs. plant, butterfly, and pupa (p.adj = 0.0295). L-phenylalanine varied between plant vs. butterfly and pupa, and between frass vs. butterfly and pupa (p.adj = 0.0372–0.0436). Linoleic acid differed between frass vs. plant, caterpillar, butterfly, and wing (p.adj = 0.00195–0.0270). Linolenic acid differed in wing comparisons (plant, butterfly, and pupa vs. wing; p.adj = 0.0116–0.0453). Oleic and palmitic acids differed between plant vs. frass (p.adj = 0.00869 and 0.00921, respectively), and phytol differed between frass vs. wing (p.adj = 0.0429). Metabolic enrichment analyses performed in MetaboAnalyst revealed significant enrichment of the pathways for biosynthesis of unsaturated fatty acids (5 hits; *p* = 8.8 × 10⁻⁸; FDR = 1.3 × 10⁻⁵) and starch and sucrose metabolism (3 hits; *p* = 2.08 × 10⁻⁴; FDR = 0.015) (Supplementary Material 2-Supplementary figure). These results indicate that the detected metabolite set is primarily associated with processes related to unsaturated fatty acid metabolism and the transformation of diet-derived carbohydrates.

## Discussion

The metabolomic differentiation observed among larvae, pupae, adults, wings, frass, and plant tissue indicates that ontogeny in *B. polydamas* is accompanied by consistent chemical reorganization. The separation detected in PCA and hierarchical clustering was supported by PERMANOVA, confirming that the differences among groups are robust and that the developmental stage acts as a structuring axis of chemical variation. Because larvae, pupae, and adults occupy distinct ecological roles and face different pressures, stage-specific metabolomic profiles likely represent adaptive reallocations associated with defense, growth, dispersal, and reproduction (Werner and Gilliam 1984; Moran 1994; Späth et al. 2022). In addition, because all individuals were maintained on a single specimen of *(A) gigantea*, this indicates that the detected patterns are more likely associated with developmental transitions and metabolic processing than with variation in host plant chemistry. Ontogenetic shifts in metabolic profiles have been reported for the damselfly *Coenagrion hastulatum* (Odonata: Coenagrionidae) (Späth et al. 2022), for the specialist caterpillar *Chlosyne lacinia* (Lepidoptera: Nymphalidae) (Gallon et al. 2024; Moreira et al. 2024), and for *Kallima inachus* (Lepidoptera: Nymphalidae) (Li et al. 2022) across different developmental stages, diapause, and stress conditions. Here, we report this pattern for the first time in *(B) polydamas* (Lepidoptera: Papilionidae). The variation observed throughout ontogeny indicates that the metabolomic profile is not static but reorganized across life stages, reflecting stage-specific patterns of metabolite retention, transformation, and allocation (Gallon et al. 2024; Moreira et al. 2024).

The clustering of pupae and adults within the same clade, together with the overlap observed in the PCA, indicates metabolic continuity during metamorphosis (Fig. 3), supporting our second hypothesis. Despite the extensive morphological reorganization that characterizes this transition, the proximity between these stages suggests that part of the metabolic pool is maintained or gradually reorganized rather than undergoing an abrupt shift between developmental phases. In chemically defended herbivores such as *B. polydamas*, this continuity may be ecologically relevant if larval-acquired metabolites, energetic reserves, or precursor compounds are preserved into adulthood, thereby reducing the costs of rebuilding defensive or physiological functions after metamorphosis (Moran 1994; Nishida 2002). A similar pattern has been reported for *Chlosyne lacinia*, where consecutive ontogenetic stages tend to resemble each other more closely than earlier stages (Gallon et al. 2024). This pattern reinforces the idea that ontogenetic complexity involves not only morphological changes but also gradual shifts in chemical profiles, reflecting underlying physiological and metabolic adjustments during development (Späth et al. 2022).

Both insect tissues and the consumed plant exhibited high levels of unsaturated fatty acids, particularly linoleic and linolenic acids. These compounds are commonly reported in Lepidoptera larvae (Guil-Guerrero et al. 2018) and are considered essential for proper development and adult emergence in several species (Vanderzant 1968; Sivapalan and Gnanapragasam 1979; Song et al. 2022). Oleic and palmitic acids were also annotated and, together with linoleic and linolenic acids, form a group of lipids widely detected in Lepidoptera and associated with key processes related to growth and developmental progression (Guil-Guerrero et al. 2018; Song et al. 2022; Soltani et al. 2024). Although these fatty acids were detected across samples, their relative abundance varied among the analyzed materials. Linoleic acid was more abundant in insect tissues and frass than in the ingested plant, with the highest relative levels observed in the excreta. This pattern may partially reflect the elimination of lipid fractions that were not fully absorbed during digestion (Addeo et al. 2024). However, it may also indicate a contribution from the symbiotic microbiota, which can influence intestinal lipid composition and potentially modulate the availability or transformation of these compounds within the host (Qian et al. 2023; Yang et al. 2025). In contrast, linolenic acid was more abundant in the wings. This distribution may indicate a degree of metabolic specialization, potentially linked to selective allocation or translocation of lipids to particular structures during development. Studies on other Lepidoptera species have shown that this fatty acid plays an important role in the transition from larval to adult stages (Guil-Guerrero et al. 2018; Wang et al. 2022a; Song et al. 2022; Soltani et al. 2024). Because butterfly wings are energetically linked to adult locomotion (Chai and Srygley 1990), the enrichment of linolenic acid in this tissue may reflect allocation to membrane composition, structural maintenance, or energy-demanding functions associated with flight, dispersal, escape from predators, and mate searching (Fraenkel and Blewett 1945; Arrese and Soulages 2010). Furthermore, enrichment analyses performed in MetaboAnalyst indicated an overrepresentation of pathways associated with the biosynthesis of unsaturated fatty acids according to KEGG annotations, reinforcing the central role of lipid metabolism in the metabolic profile observed across the analyzed materials.

The first hypothesis of this study, concerning the distinction between consumed plant material, frass, and insect tissues, was also supported by the multivariate analyses. In addition to variations in the abundance of fatty acids such as linoleic, linolenic, oleic, and palmitic acids, differences in the profile of phenolic compounds were also observed among the analyzed materials. In this context, 4-hydroxybenzyl alcohol showed greater differentiation in the frass, whereas 4-hydroxyphenylacetic acid was significantly more abundant during the larval stage. These compounds are generally associated with the shikimate pathway, a central metabolic route responsible for the synthesis of aromatic amino acids in plants, bacteria, and fungi (Dosselaere and Vanderleyden 2001; Shen et al. 2019; Valera et al. 2025; Peng et al. 2026). Metabolites derived from this pathway are frequently reported in microorganisms, particularly bacteria and fungi (Hareland et al. 1975; Douglas 2009; Engel and Moran 2013; Zhao et al. 2023; Chen et al. 2024a; Li et al. 2025; Wang et al. 2025; Chinantuya et al. 2025). However, their occurrence has also been documented in larval tissues, adult insects, and frass across different insect groups (Friedrichs et al. 2022; Pereira et al. 2025). These observations indicate that these metabolites may originate from multiple sources, including the host insect, its diet, and potentially associated microorganisms. Therefore, although microbial biotransformation in the larval gut represents one possible explanation, the present data do not allow discrimination between microbial and endogenous insect metabolism. Consequently, compounds such as 4-hydroxyphenylacetic acid and 4-hydroxybenzyl alcohol may reflect metabolic transformations occurring during digestion through host enzymatic activity, microbial metabolism, or a combination of both. Additional studies integrating metabolomics with microbiome characterization or functional assays will be necessary to clarify the contribution of gut-associated microorganisms to these metabolic transformations.

Another metabolite that varied among stages was D-fructose-6-phosphate, a central intermediate of carbohydrate metabolism that connects pathways such as glycolysis, sucrose metabolism, and trehalose biosynthesis (Campbell et al. 2014; Tauzin et al. 2019; Guo et al. 2023; Oliveira et al. 2023; You et al. 2026). In this study, this compound was more abundant in the plant, pupae, and adults, whereas relatively lower levels were detected in larvae, frass, and wings. Its presence in the plant is expected, since D-fructose-6-phosphate is directly involved in primary plant metabolism, including glycolysis and pathways associated with sugar synthesis and mobilization (Guo et al. 2022). In insects, differences among stages may reflect physiological changes during development. For example, in larvae of moth *Helicoverpa armigera* (Lepidoptera: Noctuidae), the content of glucose-6-phosphate decreases from the onset of the wandering stage to the prepupal stage (Gao et al. 2023). The higher abundance observed in pupae and adults may therefore reflect the reorganization of carbohydrate metabolism that accompanies metamorphosis (Si et al. 2023). D-fructose-6-phosphate occupies a central position in glycolysis and sugar metabolism, participating in the interconversion of glucose, trehalose, and glycogen and directly influencing cellular energy production (Campbell et al. 2014; Tauzin et al. 2019; Guo et al. 2023; Oliveira et al. 2023; You et al. 2026). During metamorphosis, substantial changes in glycolytic pathways and energy metabolism are commonly reported, accompanying the breakdown of larval tissues and the formation of adult structures (Si et al. 2023). In adults, carbohydrate mobilization is often associated with energy production and the maintenance of elevated metabolic activity (Kozhantshikov 1938; Moreira and Hermes-Lima 2024). In butterflies, as their metabolism is limited by temperature, such energetic demands are closely linked to ecologically relevant functions including sustained flight, dispersal, mate searching, territorial interactions, and predator avoidance (Boggs 2009). In contrast, larvae showed comparatively lower levels of D-fructose-6-phosphate, because this stage is characterized by rapid growth and high energetic demand (Harrison et al. 2012). This compound is likely to be rapidly used in the glycolysis, glycogen storage, or trehalose biosynthesis, thereby limiting its accumulation in larval tissues while supporting developmental processes and biomass production.

L-phenylalanine was more abundant in pupae and adults than in the other analyzed samples. This aromatic amino acid plays a central role in insect metabolism, as it serves as a precursor of tyrosine (Zografou et al. 2001; Mani et al. 2023), which is subsequently converted into catecholamines and melanin (Chen et al. 2022, 2024b). These compounds are directly involved in cuticle pigmentation and sclerotization processes that occur during metamorphosis (Wang et al. 2022b; Bai et al. 2025). The elevated levels observed in the pupal stage may therefore reflect the intense tissue remodeling and cuticle formation associated with the transition from larva to adult, during which structural integrity and protective functions are established for the adult stage. In addition, aromatic amino acids participate in the synthesis of catecholamines involved in neuromuscular regulation and physiological activity (Alcañiz and Silva 1997; Roeder 2005), which may support increased locomotor performance and behavioral activity in adults.

Phytol was also detected at higher concentrations in plant tissues and frass, which is consistent with phytol being derived from chlorophyll degradation (Islam et al. 2023). This metabolite has also been reported in digestive tract extracts and frass of herbivorous insects (Morton and Vencl 1998; Gallon et al. 2024; Dill et al. 2026). In plants, phytol and its derivatives have been implicated in signaling pathways involved in defense responses (Zhang et al. 2018; Batool et al. 2025). In addition, phytol-rich frass has been reported to deter ant predation in herbivorous beetles, suggesting that phytol may contribute to the ecological effects of frass after passing through the insect digestive tract (Morton and Vencl 1998). Therefore, phytol may play distinct ecological roles across trophic levels, acting both in plant defense and in mediating interactions between herbivores and their natural enemies (Zhang et al. 2018; Batool et al. 2025; Morton and Vencl 1998). In our study, the higher abundance of phytol in frass suggests that it may persist through digestion and contribute to frass chemical profile. As frass is deposited on leaves, flowers or on the ground (Schirmer and dos Reis 2026), phytol may also influence subsequent ecological interactions.

In spite of the clear patterns detected in the metabolomic profiles across developmental stages, some limitations of this study should be acknowledged. First, the number of biological replicates was necessarily limited due to logistical constraints and the difficulty of maintaining multiple individuals under controlled conditions throughout the entire life cycle. In addition, although GC–MS provides highly reproducible electron ionization spectra and benefits from extensive spectral libraries, only a fraction of the detected features could be confidently annotated. This limitation is likely associated with the incomplete representation of metabolites from non-model organisms, particularly insect-derived compounds and their biotransformation products, in the currently available spectral databases. Furthermore, the metabolomic profiles obtained represent only the fraction of the metabolome that is amenable to GC–MS analysis, as this approach is largely restricted to volatile or chemically derivatizable compounds. Consequently, numerous metabolites with physicochemical properties incompatible with GC–MS analysis, including non-volatile, thermolabile, or poorly derivatizable compounds, were not captured by our analytical workflow. Future studies should therefore expand sampling effort and incorporate complementary analytical platforms, such as LC–MS-based metabolomics, together with high-resolution mass spectrometry, targeted metabolite quantification, and integrative approaches combining metabolomics with transcriptomic or enzymatic data. Such efforts would provide a more comprehensive characterization of the metabolome and a deeper understanding of how host-derived metabolites are transformed, stored, or mobilized during insect development. Even with these constraints, the present study indicates that ontogeny in *B. polydamas* is not only a morphological transition but also a chemically structured process through which host-derived and endogenous metabolites are selectively retained, transformed, and redistributed, with potential implications for chemical defense strategies and ecological signaling in this aposematic species.

In conclusion, the metabolomic profiles differed consistently among plant tissues, frass, and the developmental stages of the insect, indicating pronounced metabolic reorganization throughout ontogeny. Multivariate analyses confirmed a clear separation among sample types, reflecting both physiological changes during development and the influence of plant-derived compounds on insect metabolism. Several metabolites associated with primary metabolism, including fatty acids, sugars, and amino acids, varied significantly across stages, suggesting shifts in energy allocation and biosynthetic demands. Compounds related to the shikimate pathway, such as 4-hydroxyphenylacetic acid and 4-hydroxybenzyl alcohol, showed contrasting patterns among plant material, frass, and insect tissues, indicating possible transformation or microbial contribution during digestion. Likewise, the distribution of D-fructose-6-phosphate across samples highlights changes in carbohydrate metabolism between larval growth and later developmental stages. The higher abundance of L-phenylalanine in pupae and adults further supports the role of aromatic amino acids in metamorphic processes, including cuticle formation and pigmentation. Overall, these results indicate that insect development involves substantial metabolic adjustments that reflect both endogenous physiological processes and interactions with plant-derived compounds. Understanding how these metabolites vary across developmental stages provides new insights into the biochemical dynamics underlying insect growth, metamorphosis, and plant–insect interactions.

## Supplementary Information

Below is the link to the electronic supplementary material.

Supplementary Material 1

Supplementary Material 2

## Data Availability

The data generated in this study are available in Supplementary Material 1 accompanying this article.
