# Supplementary material for: Ontogenetic shifts in the metabolomic profiles of Battus polydamas (Lepidoptera: Papilionidae: Troidini) Feeding on Aristolochia gigantea
Source: J Chem Ecol. 2026 Oct 19;52(6):96. doi: 10.1007/s10886-026-01769-5 (PMC13680301; doi:10.1007/s10886-026-01769-5)
Supplement: Supplementary file 1 [file 10886_2026_1769_MOESM2_ESM.docx]

**Supplementary material**

***Analysis and validation of the metabolic profile by GC-MS***

The efficiency of the silylation derivatization step was validated by the ubiquitous presence of the diagnostic ion *m/z* 73, corresponding to the trimethylsilyl cation [Si(CH₃)₃]⁺, confirming successful protection of polar functional groups (Kitson et al. 1996; Fiehn 2016). The recurrent detection of *m/z* 147 in carbohydrates and polyols, including Myo-inositol (RT 33.29), Maltose (RT 37.08), Sucrose (RT 45.16), and Maltotriose (RT 48.11), confirmed the presence of multiple trimethylsilylated hydroxyl groups (Fiehn 2016). In these compounds, the additional diagnostic ions *m/z* 204 and 217 further supported the identification of TMS-protected pyranose structures (Füzfai et al. 2008). The lipid and organic acid fraction was validated by the characteristic ion *m/z* 117 ([COOTMS]⁺), confirming derivatized carboxyl groups (Ribechini et al. 2011). This fragment was prominent in Palmitic acid (RT 32.55), Oleic acid (RT 37.53), Linoleic acid (RT 37.41), and Linolenic acid (RT 37.78). Phytol (RT 36.82) presented a high spectral match score (MQScore 0.94) and dominant m/z 73 fragmentation without significant *m/z* 117, consistent with its alcohol structure (Harvey and Vouros 2020). Stearic acid (RT 52.12) was identified based on its fragmentation pattern and late retention time. Amino acid metabolism was represented by L-Phenylalanine (RT 26.01) and L-Glutamic acid (RT 25.95), both with MQScore values > 0.95 and exhibiting the expected co-occurrence of *m/z* 117 and 147, consistent with simultaneous derivatization of amino and carboxyl groups (Harvey and Vouros 2020). Allantoin (RT 32.35) was detected based on its characteristic fragmentation profile. Additional metabolites included D-Fructose-6-phosphate (RT 41.58), 4-hydroxyphenylacetic acid (RT 26.21), and 4-hydroxybenzyl alcohol (RT 23.01), identified according to retention time and EI spectral matching.

**Supplementary Table 1. Molecular networking analysis of the chemical profiles (LC–MS/MS) of different developmental stages of *Battus polydamas* (caterpillar, pupa, and adult), together with frass and leaves of the host plant *Aristolochia gigantea***. Nodes represent spectral features, with size proportional to precursor ion intensity and pie charts indicating group distribution. Edges correspond to spectral similarity based on cosine scores (≥ 0.70). Catp: Caterpillar; Pp: Pupa; Bfly: Butterfly Body; Wing: Wing; Frass: Excrement; Plant: Plant.

**Supplementary Table 1. Comprehensive annotation results, spectral matching metrics, and retention time parameters for trimethylsilylated metabolites identified by GC–MS (EI)**

| **Compound name** | **GNPS Library URL** | **Instrument** | **Ion Source** | **MQ Score** | **Number of spectra** | **RT Consensus** | **RT Mean** | **Number of shared peaks** | **Smiles** | **Spectrum ID** |
| --- | --- | --- | --- | --- | --- | --- | --- | --- | --- | --- |
| Maltose | <http://gnps.ucsd.edu/ProteoSAFe/gnpslibraryspectrum.jsp?SpectrumID=CCMSLIB00005458991> | GC-EI-TOF | EI | 0,84801 | 15 | 37,08 | 37,08 | 13 | [H]OC([H])([H])C1([H])OC([H])(OC2([H])C([H])(O[H])C([H])(O[H])C([H])(O[H])OC2([H])C([H])([H])O[H])C([H])(O[H])C([H])(O[H])C1([H])O[H] | CCMSLIB00005458991 |
| Stearic acid | <http://gnps.ucsd.edu/ProteoSAFe/gnpslibraryspectrum.jsp?SpectrumID=CCMSLIB00005459042> | GC-EI-TOF | EI | 0,75751 | 18 | 52,12 | 52,12 | 10 | [H]OC(=O)C([H])([H])C([H])([H])C([H])([H])C([H])([H])C([H])([H])C([H])([H])C([H])([H])C([H])([H])C([H])([H])C([H])([H])C([H])([H])C([H])([H])C([H])([H])C([H])([H])C([H])([H])C([H])([H])C([H])([H])[H] | CCMSLIB00005459042 |
| Allantoin | <http://gnps.ucsd.edu/ProteoSAFe/gnpslibraryspectrum.jsp?SpectrumID=CCMSLIB00005458942> | GC-EI-TOF | EI | 0,848549 | 18 | 32,35 | 32,35 | 26 | [H]N=C(O[H])N([H])C1([H])N=C(O[H])N=C1O[H] | CCMSLIB00005458942 |
| Palmitic acid | <http://gnps.ucsd.edu/ProteoSAFe/gnpslibraryspectrum.jsp?SpectrumID=CCMSLIB00005459007> | GC-EI-TOF | EI | 0,849225 | 18 | 32,55 | 32,55 | 12 | [H]OC(=O)C([H])([H])C([H])([H])C([H])([H])C([H])([H])C([H])([H])C([H])([H])C([H])([H])C([H])([H])C([H])([H])C([H])([H])C([H])([H])C([H])([H])C([H])([H])C([H])([H])C([H])([H])[H] | CCMSLIB00005459007 |
| Sucrose | <http://gnps.ucsd.edu/ProteoSAFe/gnpslibraryspectrum.jsp?SpectrumID=CCMSLIB00005458844> | GC-EI-TOF | EI | 0,912791 | 18 | 45,16 | 45,16 | 25 | [H]OC([H])([H])C1([H])OC([H])(OC2(OC([H])(C([H])([H])O[H])C([H])(O[H])C2([H])O[H])C([H])([H])O[H])C([H])(O[H])C([H])(O[H])C1([H])O[H] | CCMSLIB00005458844 |
| Linoleic acid | <http://gnps.ucsd.edu/ProteoSAFe/gnpslibraryspectrum.jsp?SpectrumID=CCMSLIB00005459004> | GC-EI-TOF | EI | 0,92485 | 18 | 37,41 | 37,41 | 15 | [H]OC(=O)C([H])([H])C([H])([H])C([H])([H])C([H])([H])C([H])([H])C([H])([H])C([H])([H])C([H])=C([H])C([H])([H])C([H])=C([H])C([H])([H])C([H])([H])C([H])([H])C([H])([H])C([H])([H])[H] | CCMSLIB00005459004 |
| Oleic acid | <http://gnps.ucsd.edu/ProteoSAFe/gnpslibraryspectrum.jsp?SpectrumID=CCMSLIB00005459014> | GC-EI-TOF | EI | 0,869383 | 18 | 37,53 | 37,53 | 14 | [H]OC(=O)C([H])([H])C([H])([H])C([H])([H])C([H])([H])C([H])([H])C([H])([H])C([H])([H])C([H])=C([H])C([H])([H])C([H])([H])C([H])([H])C([H])([H])C([H])([H])C([H])([H])C([H])([H])C([H])([H])[H] | CCMSLIB00005459014 |
| Linolenic acid | <http://gnps.ucsd.edu/ProteoSAFe/gnpslibraryspectrum.jsp?SpectrumID=CCMSLIB00005459005> | GC-EI-TOF | EI | 0,835549 | 18 | 37,78 | 37,78 | 12 | [H]OC(=O)C([H])([H])C([H])([H])C([H])([H])C([H])([H])C([H])([H])C([H])([H])C([H])([H])C([H])=C([H])C([H])([H])C([H])=C([H])C([H])([H])C([H])=C([H])C([H])([H])C([H])([H])[H] | CCMSLIB00005459005 |
| Maltotriose | <http://gnps.ucsd.edu/ProteoSAFe/gnpslibraryspectrum.jsp?SpectrumID=CCMSLIB00005458876> | GC-EI-TOF | EI | 0,870944 | 18 | 48,11 | 48,11 | 19 | [H]OC([H])([H])C1([H])OC([H])(OC2([H])C([H])(O[H])C([H])(O[H])C([H])(OC2([H])C([H])([H])O[H])OC3([H])C([H])(O[H])C([H])(O[H])C([H])(O[H])OC3([H])C([H])([H])O[H])C([H])(O[H])C([H])(O[H])C1([H])O[H] | CCMSLIB00005458876 |
| 4-Hydroxyphenylacetic acid | <http://gnps.ucsd.edu/ProteoSAFe/gnpslibraryspectrum.jsp?SpectrumID=CCMSLIB00005458813> | GC-EI-TOF | EI | 0,835444 | 14 | 26,21 | 26,21 | 12 | [H]OC1=C([H])C([H])=C(C([H])=C1[H])C([H])([H])C(=O)O[H] | CCMSLIB00005458813 |
| L-Glutamic acid | <http://gnps.ucsd.edu/ProteoSAFe/gnpslibraryspectrum.jsp?SpectrumID=CCMSLIB00005458863> | GC-EI-TOF | EI | 0,823891 | 9 | 25,95 | 25,95 | 15 | [H]OC(=O)C([H])([H])C([H])([H])C([H])(N([H])[H])C(=O)O[H] | CCMSLIB00005458863 |
| L-Phenylalanine | <http://gnps.ucsd.edu/ProteoSAFe/gnpslibraryspectrum.jsp?SpectrumID=CCMSLIB00005458841> | GC-EI-TOF | EI | 0,959315 | 18 | 26,01 | 26,01 | 16 | [H]OC(=O)C([H])(N([H])[H])C([H])([H])C=1C([H])=C([H])C([H])=C([H])C1[H] | CCMSLIB00005458841 |
| D-Fructose 6-phosphate | <http://gnps.ucsd.edu/ProteoSAFe/gnpslibraryspectrum.jsp?SpectrumID=CCMSLIB00005458854> | GC-EI-TOF | EI | 0,797928 | 18 | 41,58 | 41,58 | 20 | [H]OC([H])([H])C1(O[H])OC([H])(C([H])([H])OP(=O)(O[H])O[H])C([H])(O[H])C1([H])O[H] | CCMSLIB00005458854 |
| 4-Hydroxybenzyl alcohol | <http://gnps.ucsd.edu/ProteoSAFe/gnpslibraryspectrum.jsp?SpectrumID=CCMSLIB00005458897> | GC-EI-TOF | EI | 0,947117 | 18 | 23,01 | 23,01 | 14 | [H]OC1=C([H])C([H])=C(C([H])=C1[H])C([H])([H])O[H] | CCMSLIB00005458897 |
| Phytol | <http://gnps.ucsd.edu/ProteoSAFe/gnpslibraryspectrum.jsp?SpectrumID=CCMSLIB00005458919> | GC-EI-TOF | EI | 0,942908 | 18 | 36,82 | 36,82 | 11 | [H]OC([H])([H])C([H])=C(C([H])([H])[H])C([H])([H])C([H])([H])C([H])([H])C([H])(C([H])([H])[H])C([H])([H])C([H])([H])C([H])([H])C([H])(C([H])([H])[H])C([H])([H])C([H])([H])C([H])([H])C([H])(C([H])([H])[H])C([H])([H])[H] | CCMSLIB00005458919 |

**Supplementary figure 2. Maltose.** Mirror plot of the derivatized compound obtained by GC–MS analysis (electron ionization, 70 eV), showing the comparison between the experimental mass spectrum (upper panel) and the reference library spectrum (lower panel).

**Supplementary figure 3. Maltotriose.** Mirror plot of the derivatized compound obtained by GC–MS analysis (electron ionization, 70 eV), showing the comparison between the experimental mass spectrum (upper panel) and the reference library spectrum (lower panel).

**Supplementary figure 4. Sucrose.** Mirror plot of the derivatized compound obtained by GC–MS analysis (electron ionization, 70 eV), showing the comparison between the experimental mass spectrum (upper panel) and the reference library spectrum (lower panel).

**Supplementary figure 5. D-Fructose 6-phosphate.** Mirror plot of the derivatized compound obtained by GC–MS analysis (electron ionization, 70 eV), showing the comparison between the experimental mass spectrum (upper panel) and the reference library spectrum (lower panel).

**Supplementary figure 6. L-Glutamic acid.** Mirror plot of the derivatized compound obtained by GC–MS analysis (electron ionization, 70 eV), showing the comparison between the experimental mass spectrum (upper panel) and the reference library spectrum (lower panel).

**Supplementary figure 7. L-Phenylalanine.** Mirror plot of the derivatized compound obtained by GC–MS analysis (electron ionization, 70 eV), showing the comparison between the experimental mass spectrum (upper panel) and the reference library spectrum (lower panel).

**Supplementary figure 8. Allantoin.** Mirror plot of the derivatized compound obtained by GC–MS analysis (electron ionization, 70 eV), showing the comparison between the experimental mass spectrum (upper panel) and the reference library spectrum (lower panel).

**Supplementary figure 9. 4-Hydroxyphenylacetic acid.** Mirror plot of the derivatized compound obtained by GC–MS analysis (electron ionization, 70 eV), showing the comparison between the experimental mass spectrum (upper panel) and the reference library spectrum (lower panel).

**Supplementary figure 10. 4-Hydroxybenzyl alcohol.** Mirror plot of the derivatized compound obtained by GC–MS analysis (electron ionization, 70 eV), showing the comparison between the experimental mass spectrum (upper panel) and the reference library spectrum (lower panel).

**Supplementary figure 11. Palmitic acid.** Mirror plot of the derivatized compound obtained by GC–MS analysis (electron ionization, 70 eV), showing the comparison between the experimental mass spectrum (upper panel) and the reference library spectrum (lower panel).

**Supplementary figure 12. Stearic acid.** Mirror plot of the derivatized compound obtained by GC–MS analysis (electron ionization, 70 eV), showing the comparison between the experimental mass spectrum (upper panel) and the reference library spectrum (lower panel).

**Supplementary figure 13. Oleic acid.** Mirror plot of the derivatized compound obtained by GC–MS analysis (electron ionization, 70 eV), showing the comparison between the experimental mass spectrum (upper panel) and the reference library spectrum (lower panel).

**Supplementary figure 14. Linoleic acid.** Mirror plot of the derivatized compound obtained by GC–MS analysis (electron ionization, 70 eV), showing the comparison between the experimental mass spectrum (upper panel) and the reference library spectrum (lower panel).

**Supplementary figure 15. Linolenic acid.** Mirror plot of the derivatized compound obtained by GC–MS analysis (electron ionization, 70 eV), showing the comparison between the experimental mass spectrum (upper panel) and the reference library spectrum (lower panel).

**Supplementary figure 16. Phytol.** Mirror plot of the derivatized compound obtained by GC–MS analysis (electron ionization, 70 eV), showing the comparison between the experimental mass spectrum (upper panel) and the reference library spectrum (lower panel).

**Supplementary Figure 17. Overview of enriched metabolite sets identified through metabolite set enrichment analysis using MetaboAnalyst based on pathway annotations from KEGG.** Each circle represents a metabolic pathway, where the x-axis shows the enrichment significance as −log10 (*p*-value). Circle size corresponds to the enrichment ratio, and color indicates the significance level (*p*-value). Pathways related to the biosynthesis and metabolism of unsaturated fatty acids and carbohydrate metabolism appear among the most enriched metabolic processes in the dataset.

**REFERENCES**

1. Fiehn O (2016) Metabolomics by gas chromatography-mass spectrometry: combined targeted and untargeted profiling. Curr Protoc Mol Biol 114:30.4.1–30.4.32. https://doi.org/10.1002/0471142727.mb3004s114
2. Füzfai Z, Boldizsár I, Molnar-Perl I (2008) Characteristic fragmentation patterns of the trimethylsilyl and trimethylsilyl–oxime derivatives of various saccharides as obtained by gas chromatography coupled to ion-trap mass spectrometry. J Chromatogr A 1177:183–189. https://doi.org/10.1016/j.chroma.2007.11.023
3. Harvey DJ, Vouros P (2020) Mass spectrometric fragmentation of trimethylsilyl and related alkylsilyl derivatives. Mass Spectrom Rev 39:105–211. https://doi.org/10.1002/mas.21590
4. Kitson FG, Larsen BS, McEwen CN (1996) Gas chromatography and mass spectrometry: a practical guide. Academic Press
5. Ribechini E, Bacchiocchi M, Deviese T, Colombini MP (2011) Analytical pyrolysis with in situ thermally assisted derivatisation, Py(HMDS)-GC/MS, for the chemical characterization of archaeological birch bark tar. J Anal Appl Pyrolysis 91:219–223. https://doi.org/10.1016/j.jaap.2011.02.011
